# Supplementary material for: Determining intrinsic potentials and validating optical binding forces between colloidal particles using optical tweezers
Source: Nat Commun. 2024 Feb 3;15:1020. doi: 10.1038/s41467-024-45162-w (PMC11258337; doi:10.1038/s41467-024-45162-w)
Supplement: Supplementary file 1 — Supplementary Information [file 41467_2024_45162_MOESM1_ESM.pdf]

# - Supplementary Material -
Determining intrinsic potentials and validating optical binding forces between colloidal particles using optical tweezers

Zhang et al.

Tracking bias and precision

In the following we explore the experimental determination of tracking bias and statistical noise, illustrating the process with an example involving PS particles of size 500 nanometers. As shown in Figure S1, particles A and B are adsorbed on the cover slip at the bottom interface of the sample chamber. To exclude the influence of drift and vibration in the measurement, particle A is tracked as a fiduciary marker. Using optical tweezers (point trap), a third particle C can be moved in the plane. We determine the measured position of particle B, while moving particle C towards and away from particle B, as indicated by the arrow. Since the actual position of particle B remains unchanged, the tracking bias and precision then can be obtained by analysing how the extracted position of B varies as C is moved.

In practice, we perform the following analysis: we commence with the coordinates extracted from particle tracking. To eliminate any drift, we adjust the coordinate origin to particle A. Consequently, we can determine the position of particle B when C is situated at a significant distance (e.g., 1500 nm or more). This position is referred to as the actual location of B. To calculate the bias and noise when particles B and C are at a specific center-center distance, such as e.g. $d = 550$ nm, we select all frames in which they are separated by 550 ± 5 nm. Tracking bias is calculated as the difference between the averaged position of particle B and its actual position, while precision is computed by dividing the standard deviation by $\sqrt{2}$. The extracted position of particle A also contributes to the standard deviation of the measurement, since it determines the coordinate origin. For this reason, the error must be normalized back to one particle by dividing with $\sqrt{2}$.

With our tracking method, as shown in Figure 1 a in the main text , the bias close to contact is about $\pm 2 - 3$ nm. The tracking precision (standard deviation of the statistical noise) for a single particle is better than 2 nm. When we have two mobile particles in a line trap, the precision on the extracted distance then is 2 nm $\times$ $\sqrt{2} = 2.8$ nm. Such an error associated with localisation

uncertainty is called a static error of the measurement.

Due to the limited strength of the optical trap, it is inevitable to experience some out-of-plane motion as well. We can estimate the magnitude of this motion in the z-direction to be roughly 2 times that in the y-direction (within the plane perpendicular to the laser propagation direction and in perpendicular to the line trap) (*1*). The y-motion can be readily quantified using time-resolved particle tracking and we find a 16 nm standard deviation in y motion for a particle with a diameter of 500 nm under our experimental condition. Thus, the out-of-plane motion does not exceed 35 nm. Consequently, this introduces an additional error of slightly less than 1.2 nm in distance measurement, see also ref. (*2*). Together with the tracking uncertainty of 2.8 nm, the total error (in quadrature) of the distance measured between two particles is approximately 3 nm.

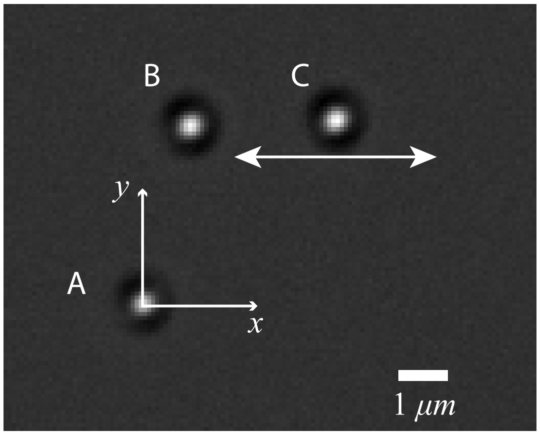

Figure S1: Illustration depicting the experimental process for determining tracking bias and precision. Particles A and B are immobile, with A establishing the coordinate system. Particle C is manipulated using optical tweezers, being brought close to and moved away from B.

Dynamic error

The dynamic error is because the camera exposure time is finite. The particle position observed from an image is time-averaged within the exposure time. From the Stokes-Einstein relation, a single particle (500 nm) within $100\ \mu$s would have a 1D motion of around 15 nm for free diffusion. Thus, the corresponding relative motion of two particles is around $\sqrt{2} \times 15 \approx 20$ nm. When these two particles are in separation smaller than 20 nm, diffusion constant decreases (*3*), resulting in a relative motion on the order of 10 nm during the camera exposure time.

These numbers are obtained based on the assumption that the movements of two particles are both free-diffusive. In our case, however, the particles are under confinement with strong attraction (for relevant measurements of depletion interactions). The relative motion is reduced due to the extra confinement from the attraction. In an extreme case of infinite attraction, the particles will be permanently bound. Therefore, their movement is fully coupled, resulting in zero relative motion. To quantify the relative motion for the depletion measurements we consider the relative motion of the particles as a function of a time lag. To show the influence of the attraction to the relative motion, results for particles with different initial distances (distance at lag-time of zero) are plotted in different panels in Figure S2. To construct the data plotted for $h = 10$ to 20 nm (Figure S2 b), we first select all the frames from the video with particle distance in between 10 nm to 20 nm. Then we take the frames which were acquired later with a time interval of τ, for all previously selected frames. Then the relative motion with lag-time τ is obtained by averaging the change of distance for all selected frames.

As shown in Figure S2, the relative motion for 2 mM F108 measurements is smaller than the 1 mM, when the initial distance is short (the range of depletion attraction is about 22 nm). This observation agrees well with the explanation that attraction slows down relative motion. As panel **b** illustrates, the relative motion in a depletant-free sample is notably greater. Extrapolating the curves (with depletion attraction) to the exposure time of $100\ \mu$s, we find that the

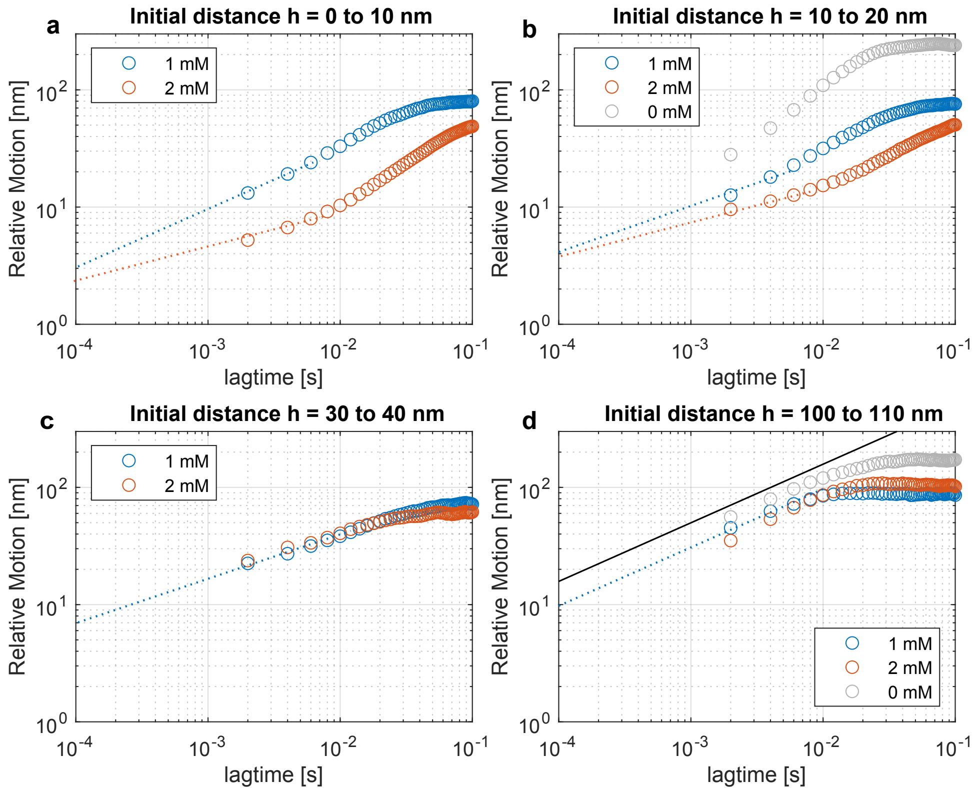

Figure S2: Relative motion plotted as a function of lag-time, for particles with different initial distances (the distance at lag-time of 0). **a** to **d**: initial distance of 0 to 10 nm, 10 to 20 nm, 30 to 40 nm and 100 to 110 nm, respectively. Gray symbols in **b** and **d** show the results of relative motion without depletion interactions. Solid line in **d** shows the prediction by the Stokes-Einstein relation. Dashed lines are guides to eyes to show the extrapolation to 100 μs. The analysis is done with the measurement of polystyrene beads (500 nm) in pluronic F108 (1 mM and 2 mM) water-based (PBS 1X) solutions.

relative motion is smaller than $4 - 5$ nm when the distance is smaller than 20 nm. The relative motion increases as the distance is increased. When the initial distance is greater than the attraction range, the difference for 1 mM and 2 mM measurements becomes small (panel **c**). The relative motion approaches the prediction by the Stokes-Einstein relation when the particles are far away (panel **d**). Since the range of interaction for our depletion interaction is about 22 nm, we take the relative motion (dynamic error) as $\Delta \simeq 5$ nm in our analysis. We neglect the small differences we observe between 1 mM and 2 mM case, or between 500 nm particles and 710 nm

particles.

We acknowledge that reducing the exposure time would decrease dynamic error, although this is currently not possible with our setup. However, such an improvement could be attainable by employing a more optimized camera and enhanced illumination conditions.

Asakura-Oosawa model comparison with experimental errors

Concerning the Asakura and Oosawa we find quantitative agreement between the experimental data and the model for the 1mM and 2mM concentration of Pluronic F108 forming micelles, as shown in Figure4 in the main text. The 2mM F108 concentration corresponds to an effective volume fraction of micelles of approximately 14%. The Asakura-Oosawa (AO) model treats the depletant particles as an ideal gas, making it accurate at low-volume fractions only. We

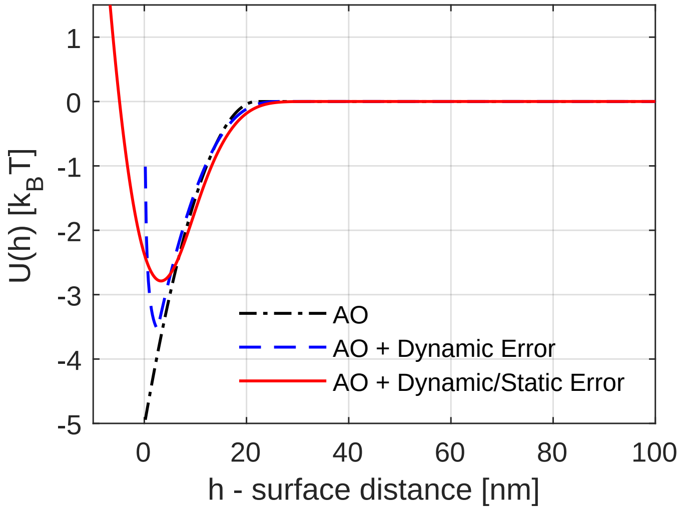

Figure S3: Prediction of depletion interaction by the Asakura-Oosawa model (AO), calculated using $U(h) = -\frac{1}{2}\pi n R(d_m - h)^2$ (dash-dotted line), where $R = 250$ nm, $n = 2.6 \times 10^4$ μm^{-3} calculated from the pluronic F108 concentration (2 mM) with aggregation number of 45. d_m is 22 nm measured by dynamic light scattering. The dashed line shows the AO model with 5 nm dynamic error. The solid line shows the AO model with 5 nm dynamic error and 3 nm static error.

also measured the depletion interactions for the sample with 3 mM of pluronic F108, for both 500 nm and 710nm particles. As depicted in Figure S4, a repulsive barrier is observable in the distance range of $20 - 35$nm. Such a repulsion barrier was previously reported in (*4, 5*) and was qualitatively explained by the layering effect of the depletant around the larger spheres. The volume fraction of the depletant with 3 mM F108 is approximately 20%. For depletant F108 micelle concentrations exceeding 3 mM, the interaction becomes so strong that particles bind 'permanently.' In such cases, we can no longer measure the potential using the Boltzmann relation due to the lack of statistics in the non-contact regime.

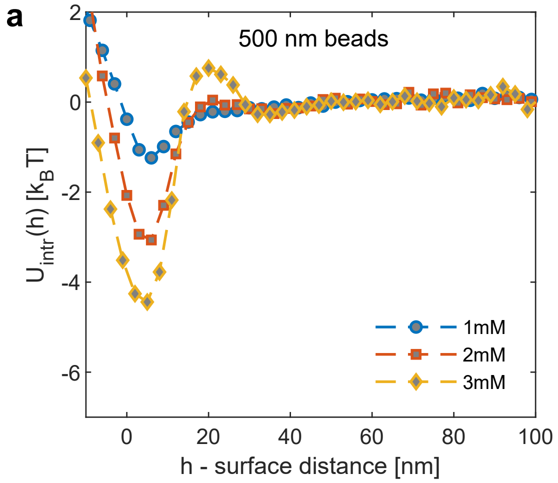

Figure S4: Depletion interaction potential measured for polystyrene (PS) beads in a pluronic F108 solution (1 mM, 2 mM and 3 mM) in a water based buffer (PBS 1X) at temperature of 40 °C. **a**, **b** PS with diameter of 500 nm and 710 nm, respectively.

Convergence of the DDA method

All DDA data shown in the main text correspond to discretized spheres with $N = 1365$ discrete dipoles per sphere. In this section, we present a brief convergence study on the optically induced interactions between the pair of particles along the line that joins the respective centers.

In Figure S5 the interaction force is shown for different levels of discretization, ranging from

$N = 251$ to $N = 1365$ dipoles per sphere. The force is computed for the line-trap illumination polarized along the x-axis (joining centers of spheres) or perpendicular to it (y-axis) as stated in the labels of each data set. As can be seen in Figure S5, the convergence in the short distance regime (from contact to 20 nm) with the number of dipoles is acceptable. The difference in force between $N = 895$ and $N = 1365$ is smaller than $\sim 4\%$ for all considered sizes, distances and polarizations.

A commonly accepted rule of thumb for estimating the minimum acceptable dipole distance in DDA is to use roughly half the cube edge. In our case, this minimum distance ranges between 18 nm and 26 nm, which is clearly above the close-to-contact distances we are interested in. However, this heuristic rule applies when the entire scatterer is substituted by a single dipole (eg. a sphere is modeled by a single dipole). In our case, each sphere is discretized and the total force is the result of the interaction among all dipoles. The contribution to the total force from pairs of dipoles in close proximity on each sphere is actually a small fraction of the total force. Hence, we estimate that our force calculations, as shown in this section, are sufficiently converged and the heuristic rule of minimum acceptable distance does not apply to such an extent that precludes a reasonable estimation of the interaction force down to contact.

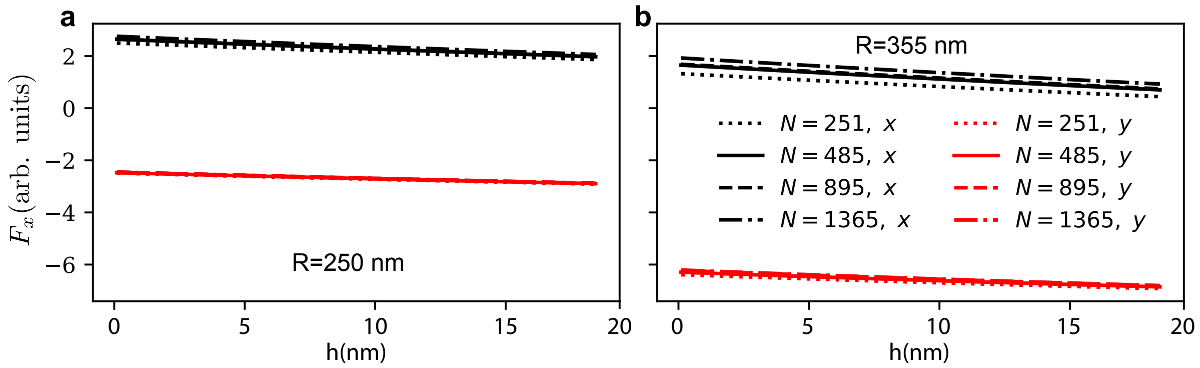

Figure S5: Convergence of the calculated interaction force by the DDA method as a function of distance and number of particles for radius $R = 250$ nm (**a**), and $R = 355$ nm(**b**) in both polarizations and for different number of dipoles per sphere.

References

1. Alexander Rohrbach. Stiffness of optical traps: quantitative agreement between experiment and electromagnetic theory. *Physical Review Letters*, 95(16):168102, 2005.

2. Paul L Biancaniello and John C Crocker. Line optical tweezers instrument for measuring nanoscale interactions and kinetics. *Review of Scientific Instruments*, 77(11), 2006.

3. T.G.M. Van de Ven. *Colloidal Hydrodynamics*. Colloid Science; 4 Professional and Technical. Academic Press, 1989.

4. John C Crocker, Joseph A Matteo, Anthony D Dinsmore, and Arjun G Yodh. Entropic attraction and repulsion in binary colloids probed with a line optical tweezer. *Physical Review Letters*, 82(21):4352, 1999.

5. Clemens Bechinger, Daniel Rudhardt, Paul Leiderer, Roland Roth, and Siegfried Dietrich. Understanding depletion forces beyond entropy. *Physical Review Letters*, 83(19):3960, 1999.
